# Contrasting snus and NRT as methods to quit smoking. an observational study

**DOI:** 10.1186/1477-7517-9-10

**Published:** 2012-02-29

**Authors:** Janne Scheffels, Karl E Lund, Ann McNeill

**Affiliations:** 1 Norwegian Institute for Alcohol and Drug Research (SIRUS), PO Box 565 Sentrum, 0105 Oslo, Norway; 2 UK Centre for Tobacco Control Studies, Division of Epidemiology & Public Health, University of Nottingham, Hucknall Road, Nottingham, NG5 1PB, UK

## Abstract

**Background:**

Snus is considerably less hazardous to health than cigarettes. Recent data from Scandinavia have indicated that many smokers use snus as a method for quitting smoking.

**Methods:**

Data from five repeated cross-sectional surveys of Norwegian men and women aged 16-74 were pooled (N = 6 262). Respondents were asked about current and former smoking and snus use. Former daily smokers (N = 1219) and current daily smokers who had tried to quit at least once (N = 1118) were asked about the method they had used at their latest quit attempt and how many quit attempts they had made. Former smokers were also requested to report what year they had made their final quit attempt.

**Results:**

Snus was the most common method used for quitting smoking among men, while NRT was most often used among women. Stratifying the data according to year of quitting smoking (1945-2007) indicated a significant increase in use of the methods for quitting asked about over time. Among men, this was largely due to an increase in the use of snus. Among male quitters under the age of 45 years, 45.8% of those who had used snus on their last attempt to quit were current non-smokers (OR = 1.61, CI 1.04-2.29), while 26,3% of those who had used NRT were current non-smokers. 59.6% of successful quitters and 19.5% of unsuccessful quitters who had used snus as a method for quitting smoking had continued to use snus on a daily basis after quitting.

**Conclusion:**

Norwegian men frequently use snus as a method for quitting smoking whereas women are more likely to use NRT. The findings indicate that switching to snus can be an effective method for quitting smoking.

## Introduction

In the European Union, with Sweden as the only exemption, snus has been banned since 1992. Norway is not a member of the EU and as such is not affected by the snus ban. Recently we have observed a significant decline in smoking prevalence in Norway, among both men and women and in all age groups. A corresponding increase in snus use has been observed, in particular among young men, where the decline in smoking has been particularly evident [1]. This resembles the situation in Sweden, where cigarette use has declined dramatically over time while snus use has been at a high level or increasing. A relationship between the increase in snus use and the decrease in smoking has been suggested [2,3], but is strongly debated [4]. Recent data from Norway has indicated that many smokers are using snus as a method for quitting [5,6]. Several studies based upon observational data have reported high success rates among men who have used snus as a method for quitting smoking. One such study was Ramstrøm & Foulds' [7] analysis of data from a cross-sectional survey made in Sweden in 2000-2001, where they found a success rate of 66% among men who had used snus as a single aid, as compared with 47% among nicotine gum users (OR 2.2, 95% CI 1.3-3.7) or 32% for those using the nicotine patch (OR 4.2, 95% CI 2.1-8.6), Giljam & Galanti [8] found in a retrospective survey of Swedish smokers and ex-smokers that having used snus at the latest quit attempt increased the probability of being abstinent by about 50% (OR 1.54, 95% CI = 1.09-2.20). In the US, Rodu & Phillips [9] compared methods used in last quit attempts from cross-sectional survey data from year 2000, and they also found higher success rates for people who used snus to quit smoking (73% of these were no longer smokers at the time of the survey) compared to people who had used nicotine gum (34% former smokers) or nicotine inhalers (28% formers smokers). A recent study among Norwegian males aged 20-50 based upon retrospective questions about use of cessation methods showed that the odds ratio for abstinence from smoking at the time of the survey was 2. 68 (p < .001) for those who had used snus compared with those who had used nicotine chewing gum [5].

In recent years a growing number of studies and systematic reviews have concluded that use of snus is substantially less hazardous than cigarette smoking [10,11]. This conclusion was also reached by the only systematic review of the evidence from studies that allow direct comparison of relative risk of smoking and snus in the same populations [12]. The magnitude of the overall reduction in hazard is difficult to estimate, but it is at least 30% for pancreatic cancer, at least 50% and probably more for oral and other gastrointestinal cancer, and possibly 100% for lung cancer and chronic obstructive pulmonary disease [10]. A study using a modified Delphi approach (judgment by a panel of experts) to estimate the relative hazard concluded that snus was likely to be at least 90% less harmful than smoking [13]. A simulation model based, among other things, on these estimates of risk showed that the switch from smoking to snus represented only a very minor difference in survival compared to smokers who gave up all use of tobacco [14]. Given the increasing evidence that snus and cigarettes have very different impact on user's health, tobacco harm reduction is increasingly seen as a promising approach to reduce the major public health problem that smoking still represents [15,16]. Harm reduction in a tobacco frame implies actively encouraging inveterate smokers to switch to safer sources of nicotine, such as e.g NRT and snus. Substituting cigarettes with NRT or snus facilitates risk reduction by allowing smokers to become smoke-free without abstaining from nicotine or tobacco respectively, but complete abstinence is still achievable [5,7,17]. There is a great deal of political controversy connected to the issue of harm reduction, also in Scandinavia, where the health authorities have stated that they do not want to recommend smokers to switch to snus or use snus as a method for smoking cessation [18].

The aim of this study was to assess the extent to which snus has been used as a method for quitting smoking among Norwegian males and females, compared to other methods such as use of NRT, according to the year the quit attempt was made. Another aim has been to study the association between methods for quitting and socio-demographic background, smoking history and current snus consumption. Finally, quit rates for smoking and continuation of snus use after quitting smoking have been investigated.

## Materials and methods

The data used for this study were drawn from a larger set of yearly representative cross-sectional surveys on tobacco behaviour conducted by Statistics Norway (SSB) on behalf of the Norwegian Directorate of Health. These surveys have been conducted every year since 1973, with a base of core questions on smoking behaviour repeated yearly. Questions on the use of methods for quitting smoking were first asked in 2003. For this study, data from 2003 to 2007 were pooled, producing a sample of 6256 respondents. The average response rate for these years was 65%. The material and methods in these surveys have previously been described by Lund and co-workers [6].

### Main outcome measures

Smoking behaviour was assessed by asking all respondents 'Do you sometimes smoke?'. The response options given were: (1) yes, every day (2) yes, sometimes but not every day or (3) no. Respondents who reported no current smoking were asked whether they had ever smoked previously on a daily or occasional basis. Snus use was assessed in an identical way, except from the question about former use where it was not differentiated between daily and occasional use.

Current smokers were asked whether they had ever tried to quit smoking, and if the answer to this was yes: 'how many times in total?'. Former smokers were asked about how many unsuccessful attempts to quit they had made before their final successful attempt, and which year they had quit. Their answers to the last question were categorized into four groups: 1945 to 1977, 1978-87, 1988-97 and 1998-2007.

All former daily smokers (N = 1219) and current daily smokers who had tried to quit at least once (N = 1118) were asked what methods they had used to support their last smoking cessation attempt. The answer options were NRT gum, NRT patches, Zyban, calling the Quitline or snus. A new variable was computed to indicate those who had not used any of these methods. The variable reporting respondents' age was divided into the age groups 15-24, 25-44 and 45-74 for this study. Education was split into three categories: low (lower secondary school), medium (upper secondary school) and high (university or college education). The variable reporting number of unsuccessful attempts to quit was computed into three values: none or one, two or three, four or more. All analyses were stratified by sex.

### Analysis

Current and former smoking and snus use status was assessed for all respondents (Table 1). Use of cessation aids among men and women, former daily smokers and current daily smokers with quit attempts was examined (Table 2). The data on use of different methods was stratified according to year of stopping smoking for former smokers, for whom this information was available (Table 3). 95% confidence intervals were calculated for all estimates. The prevalence (%) and odds ratios with 95% confidence intervals of using snus and NRT when quitting were calculated according to demographic characteristics, number of unsuccessful attempts to quit and current snus use (Table 4). In the first model, the outcome was having used snus versus reporting no use of the methods asked about, using NRT or other methods for quitting smoking. In the second model, the outcome was having used NRT versus having used other methods or none of the methods included in the study. Logistic regression was also used to study the association between snus use and use of NRT and the odds of being a former, rather than a current smoker, controlling for other factors (Table 5). For the analyses aiming to review the proportion of successes according to quit method, only respondents under the age of 45 were included. This was done to avoid including former smokers or current smokers with quit attempts in the analyses who may have made their last attempt to quit before NRT became available on the market in Norway in the mid-eighties. All the analyses were performed using SPSS.

**Table 1:** Current and former smoking and snus use in the total sample of respondents.

	Men (N = 3107)	Women (N = 3155)	Total (N = 6262)
**SMOKING**			

**Daily smoker**	22.7	22.9	22.8

**Occasional smoker**	9.5	7.8	8.6

**Former daily smoker**	22.9	16.6	19.5

**Former occasional smoker**	6.5	8.6	7.5

**Never smoker**	38.9	44.1	41.6

**(Missing)**	(3)	(3)	(6)

**SNUS USE**			

**Daily snus user**	9.2	0.4	4.8

**Occasional snus user**	6.4	1.5	3.9

**Former snus user**	8.9	2.2	5.5

**Never snus user**	75.5	95.9	85.8

**(Missing)**	(11)	(14)	(25)

Percentages.

**Table 2:** Methods for quitting smoking used at last quit attempt.

	Men		Women	
	**Current smokers who tried to quit (n = 535)**	**Former smokers** **(n = 695)**	**Current smokers who tried to quit (n = 583)**	**Former smokers** **(n = 524)**

**Snus**	17.9	14.5	2.2	1.5

**Nicotine patches**	5.2	2.2	5.5	2.9

**Nicotine chewing gum**	7.7	4.2	12.7	5.9

**Other**	5.2	2.9	5	1.9

**Used no aid**	63.9	76.3	74.6	87.8

Men and women, current smokers who tried to quit and former smokers. Percentages.

**Table 3:** Former smokers use of snus or NRT at last quit attempt, according to year of quitting smoking.

		Used no aid	Other	NRT	Snus	Total
**Men** **(N = 691)**	1945-1977	95.7 (92.34-99.08)	0	0	4.3 (0.94-7.66)	100.0 (140)
	
	1978-87	90.8 (85.37-96.23)	0	0	9.2 (3.77-14.63)	100.0 (109)
	
	1988-97	76.0 (69.97-82.93)	0,7 (-0.65-2.05)	13.7 (8.12-19.28)	9.6 (4.82-14.34)	100.0 (146)
	
	1998-2007	61.9 (56.37-67.43)	6.4 (3.61-9.19)	8.1 (4.99-11.21)	23.6 (18.76-28.44)	100.0 (296)

**Women (N = 523)**	1945-1977	100	0	0	0	100.0 (66)
	
	1978-87	98.7	0	0	1.3 (-1.25-3.85)	100.0 (76)
	
	1988-97	87.9 (82.50-93.30)	0.7 (-0.8-2.08)	10.7 (5.58-15.82)	0.7 (-0.68-2.08)	100.0 (140)
	
	1998-2007	81.2 (76.27-86.13)	3.4 (1.11-5.69)	12.9 (8.67-17.13)	2.5 (0.53-4.47)	100.0 (241)

Percentages (95% confidence intervals).

**Table 4:** Prevalence (%) and adjusted odds ratios (OR) with 95% confidence intervals (CI) of using NRT or snus on latest quit attempt among former smokers and current smokers with quit attempts (N = 2337) according to age, education, number of unsuccessful attempts to quit and current snus use

	Men (1230)		Women (1107)	
**SNUS**				

***Use of snus on latest quit attempt***	16% (197/1230)		1.9% (21/1107)	

**Age**	% (n/N)	OR* (95% CI)	% (n/N)	OR* (95% CI)

**15-24**	50 (55/110)	1.00	6 (6/100)	1.00
**25-44**	24 (87/362)	0.40 (0.20-0.80)	2.9 (13/451)	3.44 (0.60-19.95)
**45-74**	7.3 (49/674)	0.14(0.07-0.28)	0.4 (2/522)	1.23 (0.12-12.42)
**(Missing)**	(84)		(34)	

**Education**				

**Low**	15.3 (34/222)	1.00	0.6 (1/166)	1.00
**Medium**	16.3 (124/760)	0.67 (0.36-1.24)	1.7 (12/686)	1.44 (0.15-14.32)
**High**	14.6 (32/219)	0.79 (0.36-1.67)	2.6 (6/230)	1.50 (0.13-17.94)
**(Missing)**	(29)		(25)	

**Number of unsuccessful attempts to quit**				

**None or one**	13.5 (72/533)	1.00	0.7 (3/424)	1.00
**2 or 3**	19.3 (73/378)	1.27 (0.75-2.16)	2.3 (9/389)	8.99 (1.40-57.82)
**4 or more**	16.3 (52/319)	1.27(0.72-2.28)	3.1 (9/294)	0.84 (1.63-70.67)

**Current snus use**				

**No**	5.6 (59/1053)	1.00	0.8 (9/1080)	1.00
**Yes**	78 (138/177)	45.15 (28.09-72.59)	44.4 (12/27)	147.38 (32.55-667.37)

**NRT**				

***Use of NRT on latest quit attempt***	9.2% (113/1230)		13.7% (152/1107)	

**Age**	% (n/N)	OR* (95% CI)	% (n/N)	OR* (95% CI)

**15-24**	2.7 (3/110)	-	8 (8/100)	1.00
**25-44**	9.7 (35/362)	-	14.2 (64/451)	1.62 (0.71-3.76)
**45-74**	10.5 (71/674)	-**	14.9 (78/522)	1.75 (0.77-4.0)
**(Missing)**	(84)		(34)	

**Education**				

**Low**	7.7 (17/222)	1.00	15.7 (26/166)	1.00
**Medium**	10 (76/760)	1.19 (0.67-2.10)	13.1 (90/686)	0.81 (0.49-1.33)
**High**	6.4 (14/219)	0.69 (0.33-1.46)	13.5 (31/230)	0.75 (0.41-1.37)
**(Missing)**	(29)		(25)	

**Number of unsuccessful attempts to quit**				

**None or one**	5.8 (31/533)	1.00	9.4 (40/424)	1.00
**2 or 3**	9.3 (35/378)	1.53 (0.89-2.62)	12.9 (50/389)	1.48 (0.94-3.35)
**4 or more**	14.7 (47/319)	2.73(1.66-4.51)	21.3 (62/294)	2.55 (1.63-3.99)

**Current snus use**				

**No**	10.3 (108/1053)	1.00	14.3 (151/1080)	-
**Yes**	2.8 (5/177)	0.27(0.10-0.75)	0.7 (1/27)	-**

* Mutually adjusted OR's.

** Ratios not reported due to missing cases.

**Table 5:** Prevalence (%) and adjusted odds ratios (OR) with 95% confidence intervals (CI) of having quit smoking at the last attempt among men under the age of 45, according to use of snus or NRT, age and education.

	Men (N = 472)	Women (N = 551)
**SNUS**	OR (95% CI)	OR (95% CI)

**Age**		

**15-24**	1.00	1.00
**25-34**	1.91(1.12-3.55)	1.61 (0.91-2.85)
**35-44**	2.02 (1.12-3.57)	1.51 (0.87-2.61)

**Education**		

**Low**	1.00	1.00
**Medium**	1.38(0.69-2.69)	1.52 (0.71-3.23)
**High**	3.13(1.39-7.06)	3.6 (1.60-8.2)

**Use of snus on the last attempt to quit**		

**No**	1.00	1.00
**Yes**	1.61(1.04-2.49)	0.72 (0.25-2.06)

**NRT**	OR (95% CI)	OR (95% CI)

**Age**		

**15-24**	1.00	1.00
**25-34**	1.85(1.05-3.26)	1.69 (0.95-2.99)
**35-44**	1.87(1.07-3.25)	1.62 (0.93-2.8)

**Education**		

**Low**	1.00	1.00
**Medium**	1.40 (0.71-2.74)	1.45 (0.67-3.09)
**High**	3.19 (1.42-7.20)	3.41 (1.50-7.78)

**Use of NRT on the last attempt to quit**		

**No**	1.00	1.00
**Yes**	0.50 (0.23-1.09)	0.44 (0.24-0.79)

## Results

### Current and former tobacco use

The data pool contained 6262 respondents. 22.8% of these were daily smokers and 8.6% occasional smokers at the time of the interview. There were no significant gender differences in this pattern. 19.5% of all respondents reported former daily smoking, but no current smoking, while 7.5% were former occasional smokers. 41.6% had never smoked. 4,8% of all respondents used snus on a daily basis at the time of the survey, while 3,9% were occasional snus users. Snus use was most prevalent among men: 9.2% of male respondents reported daily use of snus at the time of the interview, compared to 0.4% of females (Table 1). 78.3% of all current daily smokers (N = 1430) had attempted to quit smoking at least once in their smoking career (not in table).

### Frequency of use of methods for quitting smoking

In total, about one in four former daily smokers or current daily smokers with quit attempts (ever daily smokers with quit attempts) reported use of any of the methods asked about for quitting smoking, with men being slightly more likely to have used one than women. Of the methods for quitting smoking that was asked about, snus was the most common used by men: 14.5% of all male former daily smokers and 17.9% of current daily smokers with quit attempts had used snus at their latest attempt to quit. NRT was the most common method of those enquired about used by women: 8.8% of all female former smokers and 18.8% of current smokers with quit attempts had used NRT on their latest attempt to quit. For both men and women, NRT had been used twice as often among current smokers with quit attempts as among former smokers (Table 2).

Snus had been used as an aid for cessation at the latest quit attempt by 4.3% of men who had quit before 1977, by almost 10% of men who had quit during the following two decades, and by 23.6% of the most recent quitters. Whilst the use of NRT was slightly higher than the use of snus among men who had quit between 1988-97, almost three times as many men had used snus (23,6%) than NRT (8,1%) among those who had quit the last decades. Among women, NRT was the most common method used for cessation since it was introduced on the market in Norway in the mid eighties (Table 3).

### Characteristics of respondents who had used snus or NRT to aid cessation

While 16% of all male former smokers or current smokers with quit attempts had used snus on their most recent quit attempt, only 1.9% of all female former and current smokers had done so. Around half of all male former smokers or current smokers with quit attempts between the ages of 15 and 24 (OR 1.00) had used snus to aid their last attempt to quit, one out of four between the ages of 25 and 44 (OR = 0.40, 95% CI 0.20-0.80), and only 7.3% above the age of 45(OR = 0. 14, 95% CI 0.07-0.28). 5,6% of those who were not using snus at the time of the interview (OR = 1.00) had used it to quit smoking, while 78% of all current snus users had (OR = 45,15, 95% CI = 28,09-73,59) (Table 4).

Increasing number of unsuccessful quit attempts showed a positive association with having used NRT at latest quit attempt for both men and women. While 5,8% (OR = 1.00) of male former smokers or current smokers with no or one unsuccessful quit attempts had used NRT, 14,7% (OR = 2.73, 95% CI = 1.66-4.51) of those who had tried to quit four times or more without succeeding had used this method. Among women, 9,4% (OR = 1.00) of those with zero or one unsuccessful quit attempts had used NRT, compared to 21,3% (OR = 2.55, 95% CI = 1.63-3.99) among those with four or more unsuccessful attempts. Current snus use showed a negative association (OR = 0.27, 95% CI = 0.10-0.75) with having used NRT at the last attempt to quit among men (Table 4).

### Quit rate by use of snus or NRT and continuation of snus use

Of all male ever daily smokers with quit attempts under the age of 45 in this sample who had used snus to aid their last attempt to quit smoking (N = 142), 45.8% were no longer smokers at the time of the interview. Among male ever daily smokers with quit attempts who had used NRT (N = 38), 26,3% were no longer smokers when interviewed. When analyzed in a logistic regression model controlling for age and education, the odds ratio for being a former smoker among men was 1.61 (CI 1.04-2.49) when snus had been used on the last quit attempt, compared to having used no aids or one of the other aids that was enquired about. Having used NRT showed a negative association of 0.44 (CI 0.24-0.79) with having successfully quit smoking among women. The odds of being a former smoker compared to a current smoker was more than three times higher for those with the highest level of education compared to those with the lowest, which is a stronger effect than quit method, age or sex (Table 5).

59.6% of all former smokers who had used snus as a method to quit (N = 109) used snus daily at the time of the interview, while 9.2% used snus occasionally. Of those who had used snus to try to quit smoking but had not succeeded (N = 108), 19.5% were using snus daily and 50% occasionally in addition to smoking at the time of the interview (not in table).

## Discussion

The findings from this study show that snus was used by many male smokers in Norway to help their efforts to quit smoking. Among women, NRT was the most common method used of those included in the study, while snus was not widely used. Stratifying the data according to the year of quitting smoking indicated a significant increase over time in the use of all the methods asked about to quit smoking, among both women and men. The total increase was greatest for men, and use of snus accounted for most of it. Lindstrøm [19] found the same trend in a similar analysis based on retrospective data from Sweden. In addition to gender and current snus use, age was the variable most clearly associated with using snus as a method for quitting in this study. In the youngest group of men in our sample, snus had been used in as many as half of the most recent attempts to quit, as compared to around seven per cent among men above the age of 45.

Consistent with results from other observational studies of the use of snus for quitting smoking [5-9,20] our data also indicated that snus can be an effective method to quit smoking. Among those who had quit smoking, it was more likely that on their last attempt they had used snus than NRT. One possible explanation for this could be the ability of snus to provide nicotine-addicted smokers with similar, satisfying levels of the drug. Compared to NRT, nicotine uptake from snus resembles that when smoking cigarettes [21]. In addition to snus providing nicotine in a way that is likely to satisfy the former smoker; it is also possible that snus provides some partial substitution for the sensory and social aspects of smoking. In contrast to nicotine replacement products, the use of snus may e.g be experienced by smokers as replacing some of the social functions that cigarettes had, in that that brand choice, visibility and rituals of use can represent social positioning and self presentation [22]. High consumer acceptability produced by these characteristics of snus can be some of the reason why so many Norwegian smokers prefer snus as a method for quitting, as well as for the higher quit rates for smokers who use snus as aid for quitting compared to NRT.

Our results also show that many of the smokers who quit smoking assisted by snus continued to use the product after their quit attempt. It is most likely an inevitable effect of switching to snus as a method for smoking cessation that some smokers who could have become abstinent will continue to use nicotine, but these snus users will be using nicotine in a low-risk form. Also, it should be emphasized that a considerable fraction (31.2%) of those who had used snus to quit in this study did end up completely tobacco free, as observed also in other observational studies from Scandinavia [5,7]. Still, to advise smokers to use snus as a method for quitting as a general strategy may not be sensible against this background. Another argument against this is the potential implications it may have for countries where use of smokeless tobacco is rare, or where the smokeless tobacco available is far more toxic than snus.

This study has several limitations. First, it is important to point out that our data only informs about methods used at the latest quit attempt, thus we cannot draw firm conclusions about overall changes in use of aids at quit attempts among Norwegian smokers. A possible limitation of the findings about quit attempts dating back in time, is that respondents' recall of their cessation process may be less accurate than in the in the cases where the experience is more recent. Several studies that have measured agreement between retrospective and contemporaneous reports on variables such as smoking rates [23] age of tobacco initiation [24], smoking during pregnancy [25] and levels of nicotine dependence [26], have concluded though that the validity of retrospectively reported information about smoking is acceptable. Further, the pooling of data across age groups could have introduced some uncertainty concerning how much of the age effects observed are produced by age compared to by cohort and time period. Given that the time span of the data collection is only four years, this is however not likely to be a large problem. Finally, it is important to assert the possibility that confounding factors that we have not included in the analyses may have contributed to the differences in efficacy between snus and NRT that our findings show. For example, those using snus in this study may been more quit-motivated or less dependent former smokers or different in some other way that was not measured. To explore this further, randomized controlled studies (RCT) comparing the outcome of quit attempts assisted by snus versus other methods would be helpful. To date, few RCT's have been carried out to assess the use of smoking cessation, but recently, a randomized, double-blind placebo-controlled clinical study from Serbia reported that participants who had used snus as an aid for cessation were more likely to quit smoking completely than the controls; the odds ratio (snus versus placebo) for the protocol estimates of cessation varying between 1.9 to 3.4 [27]. On the other hand, it should be noted that observational studies provide data that are superior to RCT's when it comes to evaluating effectiveness under real life conditions for different smoking cessation methods.

In conclusion, the findings of this study, considered alongside the information already published, indicate that encouraging smokers to switch to snus could be beneficial to public health. Even though there are obvious reasons to be cautious about promoting snus as a general strategy for smoking cessation, snus could be useful for smokers who are less likely than others to successfully quit, either because they are more likely to fail when they try or because they are not inclined to try.

## Competing interests

The authors declare that they have no competing interests.

## Authors' contributions

JS designed the study, performed the statistical analysis and drafted the manuscript. KEL participated in the design of the study, and helped to draft the manuscript. AMN contributed to the development of the analysis and the argumentation in the paper. All authors read and approved the final manuscript.
